# Mental health, personality, and cross-addictions as predictors of social media addiction: a machine learning longitudinal study

**DOI:** 10.1016/j.abrep.2026.100718

**Published:** 2026-06-18

**Authors:** Daniel Zarate, James Jarrad, Vasileios Stavropoulos, Connor Conkey-Morrison, Brian Hunt

**Affiliations:** School of Health and Biomedical Sciences, RMIT University, Australia

**Keywords:** Problematic social media use, Machine learning, Addiction, Coping

## Abstract

Problematic social media use (PSMU) refers to excessive, compulsive engagement with social media that impairs psychological functioning and wellbeing. Although past research has identified various correlates of PSMU, findings have been inconsistent. This study applied machine learning (ML) to predict PSMU risk over time and identify key predictors. Data were drawn from 276 adult social media users (aged 18–62) across Australia, the UK, New Zealand, and Canada. The Bergen Social Media Addiction Scale (BSMAS) was used to determine PSMU risk, applying clinical cutoffs of 19 and 24. ML models—including Random Forests, LASSO, Support Vector Machines, Logistic Regression, and Naïve Bayes—were trained using a broad set of predictors: demographic variables, personality traits, mental health indicators, motivational styles, coping strategies, and other behavioural addictions. Random Forests outperformed other models in predictive accuracy. The strongest predictors of PSMU at follow-up were baseline BSMAS scores, anxiety and COVID-related anxiety, low agreeableness, and disengagement coping (e.g., avoidance and escapism). These findings highlight the role of early symptoms, persistent anxiety, and maladaptive coping in maintaining PSMU. Future studies should incorporate additional predictors and test targeted interventions to reduce PSMU risk and promote mental wellbeing.

## Highlights

• Machine learning predicted problematic social media use across a 2-year period.
• Random Forest models outperformed other classifiers in identifying PSMU risk.
• Anxiety and disengagement coping predicted future social media addiction.
• Baseline PSMU symptoms showed strong longitudinal stability.
• Cross-addictive behaviours were weak predictors after accounting for mental health.

Social media (SM) platforms have transformed the way individuals interact, communicate, and engage with the world. With over 5 billion users globally (DataReportal, 2024), these platforms offer a range of benefits, including access to information, maintenance of long-distance relationships, and increased opportunities for self-expression. Exposure to positive content, such as motivational posts or peer encouragement, has even been linked to enhanced subjective well-being (Sun, Li, Zhang, & Zhang, 2023). However, for a growing number of individuals, social media use becomes excessive and compulsive, leading to significant distress and impaired psychological functioning. This phenomenon, known as social media addiction or problematic social media use (PSMU), has garnered increasing attention from scholars.

## 1 Conceptualising PSMU

PSMU is typically characterised by persistent, excessive engagement with social media platforms that interferes with daily responsibilities, relationships, and mental health (Kuss & Griffiths, 2017). Several frameworks have been proposed to explain the development of PSMU. Among them, motivational models suggest that individuals turn to social media when core psychological needs are unmet in their offline environment (Kuss & Griffiths, 2017). A more widely adopted model is Griffiths' Components Model of Addiction (CMA), which outlines six key features of behavioural addictions: salience, mood modification, tolerance, withdrawal, conflict, and relapse (Griffiths, 2017). The CMA has been applied extensively in addiction research, including PSMU, and forms the conceptual foundation for the Bergen Social Media Addiction Scale (BSMAS; Andreassen et al., 2016), a widely used measure in this area. Neurological research has further supported the classification of PSMU as a maladaptive use pattern, with findings indicating similarities between the brain mechanisms underlying PSMU and those associated with substance addiction (Qin et al., 2020).

While many researchers frame PSMU within the lens of addiction, others caution against overpathologising of frequent social media use (Kardefelt-Winther et al., 2017). Social media can facilitate physical activity (Goodyear, Wood, Skinner, & Thompson, 2021), improve well-being (Sun et al., 2023), and serve as an important tool for social connection. Critics argue that labelling high engagement as disordered may ignore contextual or adaptive aspects of use. Importantly, critics argue that high-frequency social media engagement may in some cases reflect coping, habit, occupational demands, or culturally normative behaviour rather than psychopathology per se (Kardefelt-Winther et al., 2017). Consequently, some scholars advocate for dimensional or problematic-use frameworks rather than strict addiction terminology. Nevertheless, PSMU has been consistently linked with poor psychological outcomes, and its predictors remain inconsistently identified in the literature.

## 2 Proposed PSMU Risk Factors

A wide range of demographic, psychological, and behavioural variables have been proposed as contributing to PSMU. These can be grouped into four conceptual clusters: demographic predictors, behavioural addictions, mental health variables, and psychological traits and coping styles.

a) **Demographic Predictors:** Studies have examined age and gender as potential risk factors for PSMU, with some suggesting that younger individuals may be more vulnerable due to identity formation and heightened social comparison (Zarate, Ball, Prokofieva, Kostakos, & Stavropoulos, 2023). Females, in particular, have shown higher prevalence of PSMU in some studies (Stănculescu & Griffiths, 2022), possibly due to more frequent social comparison and increased anxiety symptoms (Cataldo, Lepri, Neoh, & Esposito, 2021). However, large-scale meta-analyses have found inconsistent associations (Casale, Akbari, Seydavi, Bocci Benucci, & Fioravanti, 2023; Shannon, Bush, Villeneuve, Hellemans, & Guimond, 2022), suggesting that these demographic factors may not be strong standalone predictors.
b) **Behavioural Addictions (Cross Addiction):** The concept of cross addiction proposes that individuals with one form of addiction (for example, gambling, alcohol, gaming, pornography) may develop another to manage distress or replace the original behaviour (Zarate, Dorman, Prokofieva, Morda, & Stavropoulos, 2023). Empirical studies have supported links between PSMU and other behavioural or substance addictions (Zarate, Stavropoulos, Ball, de Sena, & Jacobson, 2022). This pattern, often referred to as addiction hopping, highlights the potential for shared mechanisms across behavioural addictions.
c) **Mental Health Variables:** PSMU has been consistently associated with mental health concerns, particularly anxiety, depression, and stress (Hussain & Griffiths, 2018). For instance, individuals with anxiety and depressive symptoms may use online platforms (social media or online gaming) to cope with emotional discomfort, even if the amount of time spent is similar to that of non-clinical individuals (Halaris, Hunt, Stavropoulos, Karimi, & Zarate, 2025; Kovacs, Zarate, de Sena, Tran, & Stavropoulos, 2024). The COVID-19 pandemic further exacerbated psychological distress for many, leading to increased reliance on digital platforms (Baerg & Bruchmann, 2022).
d) **Psychological Traits and Coping Styles:** Personality traits, motivational styles, and coping mechanisms have also been implicated in PSMU. Research suggests that individuals with certain personality profiles (for example, high neuroticism, low agreeableness) may be more susceptible (Huang, 2022). Maladaptive coping strategies, particularly disengagement coping (for example, avoidance and escapism), are often employed by individuals seeking to manage emotional discomfort online (Conkey-Morrison, Zarate, March, Hunt, & Stavropoulos, 2025; Halaris et al., 2025). Motivation for social media use also varies, with intrinsic and extrinsic drivers potentially influencing patterns of engagement (Halaris et al., 2025).

Informed by these domains, the current study incorporated a broad set of predictors, including demographic characteristics, mental health symptoms, personality traits, coping strategies, motivational styles, and indicators of other behavioural addictions such as problematic shopping, gambling, gaming, sexual behaviour, and substance use. These variables were selected based on their theoretical relevance and empirical links to PSMU.

## 3 Study aims and rationale

While previous research has provided useful insights, much of it relies on cross sectional data and examines predictors in isolation. This approach limits the ability to understand how multiple, interacting factors shape the development of PSMU over time. Machine learning (ML) techniques offer a promising alternative, allowing researchers to analyse large sets of interrelated variables without loss of statistical power. ML has already demonstrated utility in identifying patterns and predicting outcomes in clinical psychology and psychiatry (Shatte, Hutchinson, & Teague, 2019; Zarate, Hobson, March, Griffiths, & Stavropoulos, 2023).

Given the inconsistencies in prior findings and the need for longitudinal, multivariate analyses, the current study applies ML models to predict PSMU over time. Using a large, community-based sample and a broad range of demographic, psychological, and behavioural variables, we address the following research questions:

***RQ1***: Can machine learning models accurately predict PSMU risk?

***RQ2***: Which variables (for example, age, gender, cross addiction, depression, anxiety, stress, coping strategies, personality traits, motivational styles) best predict PSMU across time?

This study aims to inform theory and intervention by identifying key predictors of long-term PSMU risk and demonstrating the value of ML in psychological research.

## 4 Methods

### 4.1 Participants

Participants were recruited from English-speaking countries (Australia, the UK, Canada, and New Zealand) via online convenience sampling. Eligible individuals were aged 18 years or older and had at least one active social media account, regardless of usage frequency. Data were collected across three waves (2021, 2022, and 2023). The initial wave included 968 participants; after attrition, 276 participants completed all three waves and were included in the final analysis. Attrition analyses were conducted to assess differences between participants who completed the study and those who dropped out. Completers were slightly older and had lower baseline BSMAS scores than dropouts, with small effect sizes (Cohen's *d* = 0.34 and − 0.24, respectively). There were no significant differences in gender distribution (*p* = 0.092). These differences are noted in the limitations section to acknowledge potential attrition bias.

### 4.2 Procedure

Ethics approval for the original data collection was granted by the Victoria University Human Research Committee (HRE20–169) prior to commencement. Participants accessed the study online via a Qualtrics link, where they reviewed a Plain Language Information Statement (PLIS) and provided informed consent. The study was promoted through multiple channels, including social media platforms (e.g., Facebook and Reddit), the Victoria University website, digital forums, and targeted email invitations to individuals who had expressed interest in participating. Participants who did not meet the inclusion criteria were screened out and exited from the survey. Participation was voluntary, and individuals could withdraw at any time. All survey questions required a response to proceed. Sociodemographic information and baseline measures were collected at Time 1, with follow-up surveys administered at 12 and 24 months. Participants were debriefed upon completing the final wave.

### 4.3 Measures

#### 4.3.1 Addiction measures

Multiple instruments were employed to assess cross-addiction in the context of problematic social media use (PSMU). The behavioural and substance-related addictions examined included gaming, gambling, internet use, sexual behaviour, shopping, exercise, alcohol, drug, and tobacco use. To assess these, we administered the Internet Gaming Disorder Scale – Short Form (IGDS9-SF; Pontes & Griffiths, 2016), the Bergen Social Media Addiction Scale (BSMAS; Andreassen et al., 2016), the Online Gambling Diagnostic Questionnaire (OGD-Q; González-Cabrera et al., 2020), the Internet Disorder Scale – Short Form (IDS9-SF; Pontes & Griffiths, 2016), the Bergen–Yale Sex Addiction Scale (BYSAS; Andreassen, Pallesen, Griffiths, Torsheim, & Sinha, 2018), the Bergen Shopping Addiction Scale (BSAS; Andreassen et al., 2015), and the Exercise Addiction Inventory (EAI; Terry, Szabo, & Griffiths, 2004). In addition, substance-related behaviours were assessed using the Alcohol Use Disorders Identification Test (AUDIT; Fleming, Barry, & MacDonald, 1991), the Drug Abuse Screening Test (DAST-10; Cocco & Carey, 1998; Skinner, 1982), and the Cigarette Dependence Scale (CDS-5; Etter, Le Houezec, & Perneger, 2003). Detailed information on each of these measures is provided in Supplementary Table 1.

### 4.4 Personality, coping, and mental health measures

Given the established links between PSMU and psychological variables such as personality (Huang, 2022), coping behaviours (Halaris et al., 2025), and mental health conditions (Hussain & Griffiths, 2018), these constructs were also assessed in the current study. Personality traits were measured using the Ten-Item Personality Inventory (TIPI; Gosling, Rentfrow, & Swann, 2003). Motivational styles were captured with the Situational Motivation Scale (SIMS; Guay, Vallerand, & Blanchard, 2000), while coping strategies were assessed using the Brief COPE inventory (Meyer, 2001). Mental health symptoms were evaluated using the Depression Anxiety Stress Scales (DASS-21; Henry & Crawford, 2005) and the COVID Anxiety Scale (CAS; Silva, de Sampaio Brito, & Pereira, 2022). All instruments demonstrated acceptable internal consistency within the current sample (see Supplementary Table 1).

### 4.5 Statistical analysis

Data analysis, screening, assumption testing, and the development of machine learning (ML) models were conducted using RStudio (version 4.3.3) and the tidymodels framework. Problematic social media use (PSMU) was operationalised as a binary outcome (Yes/No) using two established cut-off scores on the Bergen Social Media Addiction Scale (BSMAS): a score of 19, as proposed by Bányai et al. (2017), and a stricter threshold of 24, as proposed by Luo et al. (2021). Binary classification was selected because the primary aim was to identify individuals at elevated risk of clinically meaningful PSMU rather than predict symptom severity continuously. This approach aligns with prior screening-oriented ML studies within behavioural addiction research (Stavropoulos et al., 2024). The inclusion of both cut-off scores allowed for the identification of individuals at risk of PSMU, as well as those who may meet criteria for elevated PSMU-risk. To address class imbalance in the binary outcome, the Synthetic Minority Oversampling Technique (SMOTE) was employed. SMOTE generates synthetic cases in the minority class to balance the dataset and reduce bias toward the majority class, thus improving model generalisability and predictive accuracy. However, considering recent criticisms suggesting that SMOTE may artificially inflate effect sizes or lead to overfitting (Stavropoulos et al., 2024), analyses were conducted before and after SMOTE to allow for comparison and robustness checks.

For all machine learning analyses, the dataset was randomly split into a training set (80%) and a testing set (20%). When using SMOTE, equal proportions of PSMU (Yes/No) cases were maintained within both the training and testing subsets to address class imbalance. To optimise model performance, 10-fold cross-validation and hyperparameter tuning were conducted on the training data. A minimum ratio of 50% PSMU cases was preserved across all training and validation samples, including cross-validation folds and bootstrapped subsets, to ensure consistent representation of the target class throughout model development.

The feature engineering phase involved selecting relevant predictor variables and applying a series of pre-processing procedures to prepare the data for classification. The binary outcome variable (PSMU Yes/No) was predicted using a range of features, including indicators of cross-addiction (e.g., other behavioural and substance use disorders), mental health symptoms (DASS-21), age, gender, personality traits, and coping strategies. All predictor variables were scaled and centred to ensure comparability and improve model performance. As part of the pre-processing pipeline, variables were screened for issues such as extreme skewness, zero variance, and multicollinearity. None of the predictors met the criteria for exclusion, and all were retained for analysis. Because problematic social media use demonstrates temporal stability across time, the initial machine learning models intentionally included concurrent and prior BSMAS indicators to evaluate the magnitude of autoregressive effects within classification performance. Recursive feature elimination was subsequently employed to remove BSMAS T3 and examine whether other psychosocial variables retained predictive utility beyond the autoregressive influence of prior problematic social media use symptoms. This approach was adopted to compare the relative contribution of proximal/concurrent versus more distal psychosocial indicators within the predictive framework.

Following feature engineering, several supervised machine learning algorithms recommended for binary classification were implemented. These included Random Forests, Naïve Bayes, Least Absolute Shrinkage and Selection Operator (LASSO), Support Vector Machine (SVM), and Logistic Regression (see Supplementary Table 2 for descriptions of each algorithm). Hyperparameter tuning was performed using a grid search approach within the *tidymodels* framework (Kuhn, Vaughan, & Vaughan, 2020), optimising parameters (e.g., Lambda in LASSO) to improve model accuracy.

Model performance was evaluated using key classification metrics, including accuracy, F1 score, and area under the Receiver Operating Characteristic curve (ROC-AUC). After identifying the best-performing model, a confusion matrix was generated to guide a sensitivity analysis and further evaluate predictive precision and recall.

## 5 Results

### 5.1 Participant characteristics

The demographics of the participants are presented below (see Table 1). Within the final sample, most of the participants were male (71%), employed (66.5%), and were white (69.2%). Males had a mean BSMAS score of 9.88 (SD = 4.96), with 3% scoring >24 and 7.4% scoring >19. Females had a mean score of 11.52 (SD = 5.39), with 4% scoring >24 and 12% scoring >19. Transgender/non-binary individuals had a mean score of 13.00 (SD = 5.34), none scored >24 and 20% >19.

**Table 1:** Frequencies and Descriptive Statistics of the Sample (*N* = 276).

Variables	Frequencies (%)	Mean (SD)
**Age**		31.86 (9.94); Range 18–62
**Sex**		
Female	75 (27.2%)	32.12 (10.84)
Male	196 (71%)	31.92 (9.66)
Trans/Other	5 (1.8%)	
**Employed**	183 (66.5%)	
**Race**		
White	191 (69.2%)	
Black/African American	19 (6.9%)	
Asian	51 (18.5%)	
Hispanic/Latino	11 (4%)	
Others	4 (1.5%)	
**Highest Education Level**		
Primary	3 (1.1%)	
Secondary	71 (25.7%)	
Technical	30 (10.9%)	
Some University	130 (59.1%)	
Others	5 (1.8%)	
**Relationship**		
In a relationship	117 (42.4%)	
Not in a relationship	159 (57.6%)	

### 5.2 PSMU classification and dataset preparation

Using a cutoff score of 24, the Yes/No PSMU participants were identified with No_PSMU_risk = 267 (96.74%) and Yes_PSMU_risk = 9 (3.26%). When utilising the 19-cutoff score, Yes/No to PSMU risk were identified as No_PSMU_risk = 253 (91.67%) and Yes_PSMU_risk = 23 (8.33%). To accommodate ML for RQ1, analyses were run without SMOTE, and with SMOTE to balance the dataset (i.e., Yes_PSMU_Risk = 136; 50%). Data then splits into 80% training and 20% testing in both analyses, and the proportion of Yes/No PSMU risk was significantly different for the 19 cut-offs (*χ*^2^_[1]_ = 61.31, *p <* 0.001) and the 24 cut-off scores (*χ*^2^_[1]_ = 30.70, *p* < 0.001).

### 5.3 Model performance and evaluation

To address RQ1, the prediction recipe was introduced, and 10-subdivision and bootstrapped versions of the training data were produced for cross-validation and hyperparameter tuning in the SMOTE version. Models of the Null, LASSO, SVM-Kernel, Random Forest, Naïve Bayes, and Logistic Regression in their tuned versions were introduced, trained, and tested. Table 2 summarises their performance with both cut-off scores and the use of SMOTE or not, illustrating that all models were able to perform better than the null across these conditions.

**Table 2:** Null model and tuned algorithms performance on testing data.

ML Models	ROC_AUC	PPV	F_meas	Recall	Accuracy
**Null Models**					
19 (NO SMOTE)	0.5	N/A	N/A	0	0.964
19 (SMOTE)	0.5	N/A	N/A	0	0.964
24 (NO SMOTE)	0.5	N/A	N/A	0	0.964
24 (SMOTE)	0.5	N/A	N/A	0	0.982
**Random Forest**					
19 (No SMOTE)	1	1	1	1	1
19 (SMOTE)	1	1	1	1	1
24 (No SMOTE)	1	1	1	1	1
24 (SMOTE)	1	1	1	1	1
**Logistic Regression**					
19 (No SMOTE)	0.995	0.75	0.75	0.75	0.982
19 (SMOTE)	0.995	0.60	0.66	0.75	0.973
24 (No SMOTE)	1	1	0.667	0.5	0.982
24 (SMOTE)	1	0.667	0.8	1	0.991
**LASSO**					
19 (No SMOTE)	0.995	0.75	0.75	0.75	0.982
19 (SMOTE)	1	0.6	0.667	0.75	0.973
24 (No SMOTE)	1	1	0.667	0.5	0.982
24 (SMOTE)	1	0.667	0.8	1	0.991
**Naïve Bayes**					
19 (No SMOTE)	0.895	0.222	0.308	0.5	0.919
19 (SMOTE)	0.867	0.33	0.286	0.25	0.955
24 (No SMOTE)	0.860	N/A	N/A	0	0.964
24 (SMOTE)	0.959	0	0	0	0.979
**SVM Kernel**					
19 (No SMOTE)	0.953	0.286	0.364	0.5	0.937
19 (SMOTE)	0.944	0.20	0.222	0.25	0.937
24 (No SMOTE)	0.925	N/A	N/A	0	0.964
24 (SMOTE)	0.972	1	0.667	0.5	0.991

*Note:* ROC_AUC = Receiver Operating Characteristic Area Under the Curve, PPV = Positive Predictive Value, F_meas = F measure. N/A = Not Applicable. ROC_AUC measures the model's ability to distinguish between Yes/No SMA risk cases. PPV is the proportion of true positive predictions among all positive predictions. The F measure balances precision and recall as their harmonic mean, a balance of the PPV and recall metrics. Recall measures the proportion of actual positive cases correctly identified, and accuracy represents the overall correctness of the model, including both true positive and true negative predictions.

Although the null model produced higher accuracy than some models, this metric can be misleading when there is imbalanced data (Stavropoulos et al., 2024). This is because accuracy can disproportionately reflect the model's performance on the majority class, giving a false impression of overall effectiveness while failing to adequately measure the model's ability to correctly identify instances of the minority class. When dealing with imbalanced datasets, the ROC_AUC is the most reliable metric for assessing the capacity of an ML model because it considers the trade-off between the true positive rate and the false positive rate across all possible threshold values (Richardson et al., 2024).

Among these models, Random Forests performed best, with excellent ROC_AUC, PPV, F1 score, recall, and accuracy figures. SMOTE addresses overfitting in ML models, a common issue whereby the model becomes closely aligned with the training data, failing to generalise unseen data by creating synthetic minority class examples to balance the dataset (Stavropoulos et al., 2024). Despite criticism outlined by scholars when using SMOTE, there was no difference in ML capability in random forests between using and not using SMOTE. Therefore, the SMOTE approach will be utilised because there is no inflation of results.

### 5.4 Variable importance and recursive feature elimination

**Fig. 1:**
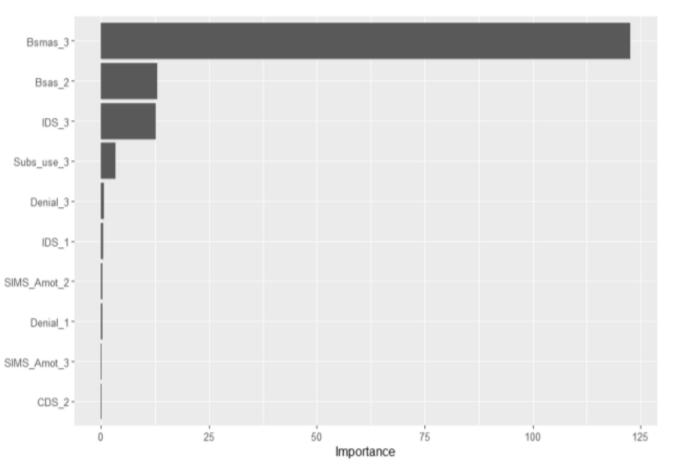
*Table of Feature Importance with all Predictors Note:* The x-axis represents the relative importance of each predictor variable within the Random Forest model, calculated using permutation-based feature importance. Higher values indicate greater contribution to the model's classification performance. The y-axis lists the predictor variables included in the model, with timepoints indicated where applicable (e.g., T1 = baseline; T3 = 24-month follow-up). BSMAS_3 = Bergen Social Media Addiction Scale at time point 3; BSAS_2 = Bergen Shopping Addiction Scale at time point 2; IDS_3 = Internet Disorder Scale at time point 3; Subs_use_3 = Brief COPE Substance use at time point 3; SIMS_Amot_2 = Amotivated motivation styles as measured by the Situational Motivational Scale at time point 2; CDS_2 = Cigarette Dependence Scale at time point 2.

To address RQ2, the recipe was adjusted to examine which variables demonstrated the most predictability of PSMU. Random Forest illustrated the most learning potential compared to the other ML models, making it the choice for further examination in determining significant variables. The first step was to compare which cutoff score would accurately predict PSMU. Random Forest demonstrated perfect capabilities (ROC_AUC = 1) without removing any predictors in predicting PSMU (see Table 3). When examining the variable importance analysis (i.e., using a permutation test to identify variable importance to sustain the observed results in 1000 permutations), it was apparent that BSMAS scores captured at T3 illustrated the most importance of predicting PSMU (see Fig. 1). While the ROC_AUC of 1.0 may appear unusually high, this model was tested on a completely unseen 20% hold-out sample to guard against overfitting. In addition, 10-fold cross-validation with 1000 bootstrapped resamples was employed during training to ensure model generalisability. When predictors were reduced using recursive feature elimination, model performance remained strong (though below perfect), supporting the robustness of the findings and reducing the likelihood of overfitting.

**Table 3:** Random Forest Model's Performance Across Different Recipes and Cutoffs.

Model	ROC_AUC	PPV	F_Meas	Recall	Accuracy
Cutoff 24 - With Only BSMAS 3	1	1	1	1	1
Cutoff 24 - Without BSMAS 3	0.872	0	0	0	0.964
Cutoff 19 - With Only BSMAS 3	1	1	1	1	1
Cutoff 19 - Without BSMAS 3	0.909	0.286	0.364	0.5	0.937
Cutoff 19 - With Top 5 Predictors	0.874	0.118	0.190	0.5	0.847

**Fig. 2:**
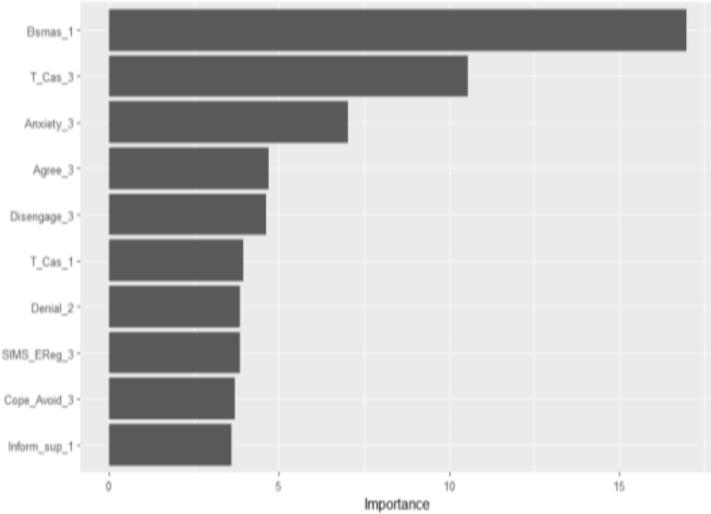
*Table of Feature Importance After Removing BSMAS at T3.Note:* The x-axis represents the relative importance of each predictor variable within the Random Forest model after removal of concurrent BSMAS T3 scores, calculated using permutation-based feature importance. Higher values indicate greater contribution to classification accuracy. The y-axis lists the remaining predictor variables included in the reduced model, with timepoints indicated where applicable (e.g., T1 = baseline; T3 = 24-month follow-up). BSMAS_1 = Bergen Social Media Addiction Scale at time point 1; T_Cas_1 = COVID anxiety scale at time point 1; Anxiety_3 = Anxiety measure included in the DASS21 at time point 3; Agree_3 = Agreeableness at time point 3; Disengage_3 = Maladaptive strategy (disengagement) as measured by the Brief COPE at time point 3; SIMS_EReg_3 = Extrinsic regulation as measured by the Situational Motivational Scale at time point 3; Cope_Avoid_3 = Maladaptive strategy (avoidant) as measured by the Brief COPE at time point 3; Inform_sup_1 = Adaptive strategy (information support) as measured by the Brief COPE at time point 1.

The next analytical step involved removing BSMAS at T3 from the predictive recipe to examine whether psychosocial variables retained predictive utility beyond the autoregressive effect of concurrent problematic social media use symptoms. This recursive feature elimination approach was intentionally implemented to compare the relative contribution of proximal/concurrent versus more distal psychosocial indicators. Upon completing this, both models (19 and 24 cut-offs) performed well (see Table 3). However, given that the 24-cut-off model showed poor PPV and F measure (i.e., 0) when removing BSMAS T3, the next step of recursive elimination exclusively focused on the 19-cut-off model. The number of variables in the recipe was then reduced to 5 by examining the new table of performance (see Fig. 2), which found that the variables that best predicted PSMU include BSMAS T1, CAS T3, anxiety T3 (i.e., anxiety was extracted from the A component of the DASS-21), agreeableness T3 (within the TIPI), and disengagement/avoidant behaviours T3 (captured by the brief COPE).

## 6 Discussion

This longitudinal study used a large, community-based sample of social media (SM) users to train machine learning (ML) models to predict individuals' risk of problematic social media use (PSMU). Risk prediction was based on multiple factors including age, gender, psychological distress, coping strategies, personality traits, and indicators of cross-addiction. Five widely used ML algorithms, with tuned hyperparameters to optimise model learning, were evaluated. Random Forests emerged as the best-performing model. Recursive feature elimination was then used to identify the strongest predictors of PSMU, with baseline BSMAS scores and anxiety symptoms identified as key indicators.

### 6.1 Accuracy of machine learning models

All ML models outperformed the null model, demonstrating strong predictive capacity. These findings align with previous psychological research showing that large, multivariable datasets can improve outcome prediction when analysed using ML techniques (Shatte et al., 2019). Notably, the use of longitudinal data, incorporating repeated measures over time, likely contributed to the high classification accuracy observed. This supports the utility of biopsychosocial and ecological models of human development (Anderson, Steen, & Stavropoulos, 2016), which highlight the dynamic interplay of individual, psychological, and contextual influences on behavioural outcomes. Compared to traditional univariate approaches, ML methods offer a more integrated and nuanced understanding of risk. Among the algorithms tested, Random Forests produced the strongest model fit, reinforcing its utility in applied psychological research. Although the Random Forest model achieved an AUC of 1.0 in one condition, several safeguards were implemented to reduce overfitting risk, including hold-out testing and follow-up models using fewer predictors (i.e., recursive feature elimination) that maintained high accuracy. This indicates the model was not overfit to the training data. Importantly, comparable model performance was observed in models with and without SMOTE, suggesting that findings were not solely attributable to synthetic oversampling procedures.

### 6.2 Key predictors of problematic social media use

Recursive feature elimination identified baseline BSMAS scores as the most salient predictor of future PSMU risk. This finding supports the reliability and predictive validity of the BSMAS as a tool for identifying problematic patterns of SM use (Gomez, Zarate, Brown, Hein, & Stavropoulos, 2024; Stănculescu & Griffiths, 2022; Zarate, Ball, et al., 2023). Longitudinal studies have similarly found that BSMAS scores remain stable over time (Santini et al., 2024; Tullett-Prado, Doley, Zarate, Gomez, & Stavropoulos, 2023), further supporting its clinical relevance for early identification and intervention. While the strong predictive value of BSMAS at Time 1 is consistent with expectations and prior research, its predictive power also underscores the potential for early identification and long-term risk tracking. However, this finding alone may not offer actionable insight for prevention or early intervention. Importantly, when BSMAS at T3 was removed from the model, other meaningful predictors emerged, namely disengagement coping styles, and low agreeableness. Some identified variables, particularly anxiety symptoms and disengagement coping, may represent modifiable intervention targets. In contrast, low agreeableness may reflect a broader dispositional vulnerability associated with interpersonal difficulties and maladaptive online engagement patterns. The absence of effects for other personality traits may suggest that interpersonal and social-relational tendencies captured by agreeableness are more relevant to problematic social media engagement than broader dispositional characteristics in this adult sample. Demographic variables such as age and gender did not emerge as strong predictors once broader psychological and behavioural variables were considered. This aligns with recent findings suggesting that demographic effects may be comparatively weak relative to psychological vulnerabilities and coping processes (Casale et al., 2023; Shannon et al., 2022).

Anxiety symptoms also emerged as robust predictors of PSMU. Both generalised anxiety (measured via the DASS-21) and COVID-specific anxiety (measured via the CAS) were associated with increased risk. The timing of the first data wave, which coincided with the height of COVID-19 restrictions, may have contributed to elevated anxiety and a greater reliance on SM as a coping mechanism (Baerg & Bruchmann, 2022). Psychological theories suggest that individuals may turn to SM to alleviate distress, but over time this reliance may reinforce maladaptive patterns of engagement, leading to increased psychological harm (Halaris et al., 2025; Serafini et al., 2020). Disengagement behaviours, such as avoidance and escapism, also played a meaningful role in PSMU. These behaviours may reflect attempts to avoid stressors or uncomfortable emotions, with social media providing an accessible and rewarding escape (Zarate, Dorman, et al., 2023). This aligns with addiction literature suggesting that individuals often turn to compulsive behaviours to regulate negative affect. Over time, reliance on SM as a means of emotional avoidance may reinforce addictive engagement patterns.

Importantly, the present findings should not be interpreted causally. Several of the strongest predictors identified in the final models, including anxiety symptoms, disengagement coping, and agreeableness, were measured concurrently with the outcome variable at T3. Their inclusion was intentional, as the study aimed to compare whether proximal/concurrent experiences or more distal psychosocial indicators demonstrated stronger predictive relevance for problematic social media use classification. Consequently, the findings are best interpreted as identifying associated risk indicators within a longitudinal machine learning framework rather than establishing causal pathways. Future studies incorporating broader contextual, developmental, and environmental variables may help clarify causal mechanisms underlying problematic social media use over time.

Interestingly, the current findings did not support the cross-addiction hypothesis. Although previous research has shown that PSMU can co-occur with other addictive behaviours (Zarate, Ball, Montag, Prokofieva, & Stavropoulos, 2022), no strong associations were found in the present study. One potential explanation may lie in the highly individualised and algorithm-driven nature of SM, which fosters persistent engagement through personalised content (Eg, Demirkol Tønnesen, & Tennfjord, 2023). These mechanisms may suggest that problematic social media engagement operates through partially distinct reinforcement mechanisms compared to some other behavioural addictions (Kuss & Griffiths, 2017).

### 6.3 Implications and future directions

Findings suggest that targeted interventions addressing anxiety and disengagement coping could be effective in reducing PSMU. Cognitive Behavioural Therapy (CBT), which aims to restructure maladaptive thought patterns and build healthier coping strategies, has shown efficacy in treating PSMU (Plackett, Blyth, & Schartau, 2023). Additionally, digital detox programs that emphasise positive reinforcement strategies and alternative sources of reward may help mitigate escapist engagement (Anandpara et al., 2024). Clinicians should consider routinely screening for SM-related problems, particularly in individuals presenting with high anxiety or avoidant coping, and especially among younger users (Stănculescu & Griffiths, 2022). Additionally, considering the high autocorrelation of PSMU, digital phenotyping methods surveying online behaviour should be considered to prevent and address this problematic (Zarate, Hobson, et al., 2023; Zarate, Stavropoulos, et al., 2022). These findings highlight several actionable targets for intervention. Even in the absence of elevated baseline BSMAS scores, individuals reporting high anxiety, disengagement-based coping, and lower agreeableness may be at elevated risk of developing PSMU. As such, clinicians and digital wellbeing interventions should prioritise identifying and addressing these psychological risk factors before problematic usage becomes entrenched.

Although the current study included a wide range of psychological and behavioural variables, future research could expand predictive models to include additional constructs such as fear of missing out (FOMO), which has demonstrated strong associations with PSMU. FOMO may drive compulsive engagement with SM platforms by heightening sensitivity to missed social opportunities, thus reinforcing problematic patterns of use. Including such variables in future ML models could improve predictive performance and help identify more nuanced user subgroups.

### 6.4 Limitations

While these findings underscore the utility of ML approaches in identifying problematic social media use (PSMU) risk, several limitations warrant consideration. First, the absence of standardised diagnostic criteria for PSMU may limit comparability across studies, as operational definitions and cut-off scores vary considerably within the literature. Additionally, the study relied exclusively on self-report measures, which are susceptible to recall and social desirability biases.

Several predictors also demonstrated poor internal consistency, particularly the OGD-Q and CDS-5. These measures were retained because they represented theoretically relevant candidate predictors within an exploratory ML framework and were not interpreted as standalone diagnostic indicators. Importantly, the lowest-reliability measures did not emerge as central predictors in the final reduced model, reducing the likelihood that they substantively shaped the primary conclusions. Nevertheless, low reliability may attenuate associations and introduce instability into variable importance estimates. Future studies should replicate these findings using more psychometrically robust measures of gambling, cigarette dependence, and COVID-related anxiety.

The relatively small number of high-risk PSMU cases may have further reduced model stability and limited the robustness of minority class classification. Although SMOTE procedures and hold-out validation were implemented to minimise these concerns, future studies with larger clinical samples should compare binary classification and continuous prediction approaches.

It is also important to acknowledge the contextual influence of the COVID-19 pandemic, during which social media functioned as a primary tool for communication, coping, and social connection. Consequently, elevated BSMAS scores during this period may not always reflect enduring addictive behaviour, but rather temporary adaptation to social isolation and environmental stressors.

Finally, the use of online convenience sampling and attrition across waves may limit generalisability. The final longitudinal sample was disproportionately male and White, and recruitment procedures may have preferentially attracted highly digitally engaged participants. Although attrition-related differences in age and BSMAS scores were relatively small, caution should be exercised when interpreting the broader applicability of the longitudinal findings. Furthermore, while all participating countries were English-speaking and shared broadly similar digital infrastructures, cultural differences in social media norms and mental health perceptions may still have influenced participant responses.

## 7 Conclusion

Despite these limitations, the findings demonstrate the promise of ML techniques in capturing complex psychological processes underlying PSMU. The stability of BSMAS scores across time, combined with the predictive value of anxiety and disengagement coping, offers a compelling case for continued exploration of ML in this domain. Future work should continue refining models by incorporating diverse samples, broader psychosocial variables, and context-sensitive designs. Ultimately, this research advances our understanding of PSMU and offers practical insights for clinical intervention and public health.

## Authors' contribution

DZ contributed to the article's conceptualization, data curation, formal analysis, methodology, project administration, and writing of the original draft.

JJ, and BH contributed to the article's conceptualization, data curation, writing of the original draft, formal analysis, methodology, and writing of the original draft. VS and CCM contributed to the final revision of the manuscript.

## Ethical Standards – Animal Rights

All procedures performed in the study involving human participants were in accordance with the ethical standards of the institutional and/or national research committee and with the 1964 Helsinki declaration and its later amendments or comparable ethical standards. This article does not contain any studies with animals performed by any of the authors.

## Confirmation Statement

Authors confirm that this paper has not been either previously published or submitted simultaneously for publication elsewhere.

## Copyright

Authors assign copyright or license the publication rights in the present article.

## CRediT authorship contribution statement

**Daniel Zarate:** Writing – review & editing, Writing – original draft, Visualization, Supervision, Software, Resources, Project administration, Methodology, Investigation, Formal analysis, Data curation, Conceptualization. **James Jarrad:** Writing – original draft. **Vasileios Stavropoulos:** Writing – review & editing. **Connor Conkey-Morrison:** Writing – review & editing. **Brian Hunt:** Writing – review & editing.

## Informed consent

Informed consent was obtained from all individual participants included in the study.

## Funding

No funding was received.

## Declaration of competing interest

The authors declare that they have no known competing financial interests or personal relationships that could have appeared to influence the work reported in this paper.

## Data Availability

Data will be made available on request.
