# Supplementary material for: Mental health, personality, and cross-addictions as predictors of social media addiction: a machine learning longitudinal study
Source: Addict Behav Rep. 2026 Jun 18;24:100718. doi: 10.1016/j.abrep.2026.100718 (PMC13312529; doi:10.1016/j.abrep.2026.100718)
Supplement: Supplementary file 1 — Supplementary material [file mmc1.docx]

**Supplementary Table 1**

**Description of Each Measure and Reliability Analyses**

| Measure | Description | α | ω |
| --- | --- | --- | --- |
| IGDS9-SF Pontes & Griffiths, 2016 | Video game addiction was measured by utilising the Internet Gaming Disorder Scale-Short-Form. The IGDS9-SF is a 9-item self-report questionnaire, where each item represents the nine criteria that define IGD. This measure assesses over 12 months the severity of a person’s gaming tendencies (e.g., “Do you feel preoccupied with your gaming behaviour?”). Each item is rated on a 5-point Likert scale, which ranges from (1 ‘Never’) to (5 ‘Very often’), with scores ranging from 9-45. Higher scores in this measure indicate greater severity of gaming addiction. | 0.74 | 0.75 |
| OGD-Q González-Cabrera et al. 2020 | Measuring the severity of an individual’s addiction to online gambling was conducted by using the Online Gambling Diagnostic Questionnaire. This measure contains 11 items, with each item representing the DSM-5 criteria for gambling disorder. Items are scored on a 5-point Likert scale, prompting participants to rate how often they engage in gambling-related behaviours, ranging from (1 ‘ Never’) to (5 ‘Very Every day’). One example item is (“Do you feel the need to spend more and more money to get the high you desire?”). Scores are summed, ranging from 11-55, with higher scores indicating greater severity. The OGD-Q also has an additional final question (item # 12) that requires respondents who report at least one behaviour to indicate how long they had been performing this behaviour in terms of less than a month, for more than a month, for more than six months, and more than 12 months. | 0.27 | 0.36 |
| BYSAS Andreassen et al. (2018) | The Bergen-Yale Sex Addiction Scale is a 6-item questionnaire measuring a person’s severity of their addiction to sex. Each item in the BYSAS represents an element within the addiction components model. This measure prompts individuals to assess over 12 months (e.g., “How often during the past year have you used sex/masturbation to forget about/escape from personal problems?”). Scores are summed, ranging from 0-24, with heightened scores indicating greater sex addiction. These items are scored on a 5-point Likert scale (0 ‘very rarely’) to (4 ‘very often’). | 0.83 | 0.84 |
| BSAS Andreassen et al. 2015 | An individual’s addiction to shopping was assessed using the Bergen Shopping Addiction Scale. The BSAS is a 7-item questionnaire, with each item representing a component within the components model of addiction and an element of presenting issues (e.g., “I think about shopping/buying things all the time.”). Scores are rated on a 5-point Likert scale (1 ‘strongly disagree’) to (5 ‘strongly agree’), ranging from 7-35, with higher scores indicating more significant shopping addiction tendencies. One example of an item is (“I think about shopping/buying things all the time.”). | 0.74 | 0.75 |
| EAI Terry et al. 2004 | The Exercise Addiction Inventory (EAI) assessed the severity of addictive exercise behaviours. The EAI is a 6-item measurement, with each item representing a component within the components model of addiction. These items are rated on a 6-point Likert scale (1 ‘strongly disagree’) to (6 ‘strongly agree’), with scores then summed, ranging from 6 to 36. One example of an item is “Exercise is the most important thing in my life.” Higher scores from this measure indicate a more significant risk of addictive exercise behaviours. | 0.77 | 0.78 |
| AUDIT Fleming et al. 1991 | To evaluate addictive an individual’s addiction to alcohol, the Alcohol Use Disorders Identification Test was utilised. This 10-item questionnaire prompts individuals to evaluate their behaviours related to alcohol consumption within the last year (e.g., “How often during the last year have you had a feeling of guilt or remorse after drinking?”). This measure uses a 5-point Likert scale, ranging from (0 ‘Never’) to (4 ‘Daily or almost daily’), with scores ranging from 0-40. Heightened scores on this measure indicate greater severity in problematic and addictive drinking behaviours. |  |  |
| DAST-10 Cocco and Carey, 1998 | The Drug Abuse Screening Test was used to measure the presence of drug addiction. The DAST-10 is a 10-item self-report measure that prompts individuals to respond ‘Yes’ or ‘No’ to statements about their drug use (e.g., “Have you had "blackouts" or "flashbacks" as a result of drug use?”). A 2-point Likert scale is utilised in this study (0 ‘No’) to (1 ‘Yes), with total scores ranging from 0-10 after being summed. Greater scores indicate a heightened risk of some form of drug addiction. | 0.78 | 0.78 |
| CDS-5 Etter et al., 2003 | The Cigarette Dependence Scale, a 5-item short version (CDS-5), assessed cigarette dependence. The CDS-5 includes five self-report items covering various aspects of smoking behaviour (e.g., "After a few hours without smoking, I feel an irresistible urge to smoke"). Participants respond using different scales depending on the question. For example, addiction is rated on a scale from 0 to 100, where 0 indicates no addiction, and 100 indicates extreme addiction, with responses recoded into categories from 1 to 5. Other questions address the number of cigarettes smoked per day, the time before the first cigarette after waking, and the perceived difficulty of quitting smoking. Scores range from 5 to 25, with higher total scores indicating a greater level of cigarette dependence​ | 0.20 | 0.38 |
| IDS9-SF Pontes & Griffiths, 2016 | Internet addiction was measured using the Internet Disorder Scale - Short Form. The IDS9-SF is a 9-item questionnaire, with each item addressing the criteria for an internet gaming disorder but tailored for this addiction. For scoring, this measure uses a 5-point Likert scale, (1 ‘Never’) to (5 ‘ Very often’) when assessing the consistency in internet usage habits (e.g., “Do you go online to escape or feel better [e.g., helplessness, guilt, anxiety]”). The total score for this measure can range from 9-45 once all items are summed up, with greater scores indicating a greater risk of this behavioural addiction | 0.70 | 0.74 |
| TIPI Gosling et al. 2003 | The Ten-Item Personality Inventory (TIPI) is a brief self-report measure designed to assess the Big Five personality dimensions: Extraversion, Agreeableness, Conscientiousness, Emotional Stability, and Openness to Experience. It consists of 10 items, with participants rating themselves on a 7-point Likert scale (1 = Disagree strongly, 7 = Agree strongly). Each personality dimension is represented by two items, one positively keyed and one negatively keyed. The TIPI is helpful for situations requiring quick assessment of personality traits. | 0.60 | 0.70 |
| SIMS Guay & Blanchard, 2000 | The Situational Motivation Scale (SIMS) is a 16-item measure designed to assess individuals' motivation in specific contexts, such as completing a project or participating in an activity. Each item is rated on a 7-point Likert scale (1 = corresponds not at all, 7 = corresponds precisely). The scale evaluates four types of motivation: intrinsic motivation (e.g., "Because this activity is fun"), identified regulation (e.g., "Because I am doing it for my good"), external regulation (e.g., "Because I have to"), and motivation (e.g., "I do not see what this activity brings me").  Scores for each motivation type are calculated by summing specific item responses. The Self-Determination Index (SDI) is also computed using the formula SDI = (2 x IM) + IR – ER – (2 x AM). Higher SDI values indicate a greater degree of self-determined motivation. | 0.71 | 0.82 |
| COPE, Meyer, 2001 | The Brief COPE Inventory is a 28-item self-report questionnaire used to assess various coping strategies individuals use in response to stress. It includes 14 subscales, each with two items, representing strategies such as Active Coping, Planning, Use of Emotional Support, Humour, and Substance Use. Participants rate each item on a 4-point Likert scale (1 = "I have not been doing this at all" to 4 = "I have been doing this a lot"). Higher scores in specific subscales reflect greater use of those coping strategies. | 0.87 | 0.90 |
| DASS-21 Henry & Crawford, 2005 | The Depression Anxiety Stress Scales - 21 is a 21-item self-report questionnaire designed to measure three distinct dimensions of emotional distress: Depression, Anxiety, and Stress. Each scale contains seven items rated on a 4-point Likert scale (0 = "Did not apply to me at all" to 3 = "Applied to me very much or most of the time"). Scores for each dimension are summed and multiplied by 2 for final scores, with higher scores indicating more severe symptoms. It assesses experiences like difficulty relaxing, nervous energy, and feelings of worthlessness. | 0.89 | 0.94 |
| CAS: Silva et al. 2022 | An individual’s fear and apprehension of COVID-19 was captured using the COVID anxiety scale. The CAS is a 7-item questionnaire that prompts people to respond to how often in the last two weeks they displayed behaviour that relates to fearing COVID-19 (e.g., ‘I have trouble relaxing when I think about COVID-19’). A 5-point Likert scale (0 ‘not at all’) to (4 ‘nearly every day over the last two weeks’), where the sum of each item is combined, with greater scores indicating greater fear of COVID-19 and ranging from 0-28. | 0.56 | 0.61 |
| BSMAS Andreassean et al. (2016) | The severity of an individual’s addiction to social media was assessed using the Bergen social media addiction scale. BSMAS is a 6-item questionnaire, with each item representing the 6-component model of addiction (e.g., “You spend a lot of time thinking about social media or planning how to use it?”). On a 5-point Likert scale, participants rate the frequency of their behaviours and attitudes toward social media usage, ranging from 1 (very rarely) to 5 (very often). Scores are then summed and can range from 6 to 30. | 0.72 | 0.74 |

**Supplementary Table 2**

**Description of Machine Learning Models and Their Hyperparameters**

| Machine Learning Model | Description | Hyperparameters | Rstudio Package |
| --- | --- | --- | --- |
| Naïve Bayes | Naïve Bayes is a simple yet effective probabilistic classification algorithm based on Bayes’ Theorem. It operates under the assumption that features are independent of each other, making it particularly fast and efficient for large datasets. Despite its simplicity, Naïve Bayes can perform surprisingly well in tasks like spam detection and text classification, where this assumption holds approximately true. | Smoothness = In Naive Bayes, smoothness refers to how the kernel adjusts the density estimates of continuous predictors. Smoothing out the estimates helps the algorithm represent the data more quickly and accurately.  Laplace = Laplace smoothing is used to avoid zero probabilities for unseen data. Adding a small constant to all counts ensures that every category has a non-zero probability, making the model more reliable and robust. | naivebayes |
| Random Forests | Random Forest is an ensemble learning method used for classification and regression. It builds multiple decision trees, each trained on different subsets of the data, and combines their predictions. This approach reduces overfitting and improves accuracy by averaging the results of individual trees. Due to their robustness and scalability, Random Forests are versatile and widely used for various predictive tasks. | min_n = In Random Forests, the min_n is the minimum value, number, or integer required for the decision tree to continue splitting. If a node has fewer than this number of data points, it will not be split further, which helps prevent overfitting and ensures that splits are meaningful.  mtry = mtry establishes how many independent variables are selected randomly and considered at each split in the decision tree. This helps improve the diversity and performance of the model by reducing overfitting. | ranger |
| Logistic Regression | Logistic Regression is a classification algorithm used to model the probability of a binary outcome. It applies a logistic function to estimate probabilities, classifying data points into two categories. This method is straightforward and interpretable, often applied in healthcare and social sciences for binary classification problems. | penalty = In logistic regression, the regularisation penalty hyperparameter aims to address generalisation errors and reduce overfitting risks. It does this by adding a cost to the model for being too complex, which encourages simpler models more likely to perform well on new, unseen data.  mixture = Ranging from 0 to 1, the mixture hyperparameter balances LASSO and ridge regression. A value of 0 represents ridge regression, shrinking coefficients but not entirely removing them, and 1 represents LASSO, where coefficients are zeroed out. | glm |
| SVM Kernel | Support Vector Machines (SVM) are classification models that aim to find the hyperplane that best separates data into distinct classes. Using kernel functions allows SVMs to solve non-linear problems by mapping data into a higher-dimensional space where linear separation is possible. This makes SVMs highly effective for complex classification tasks. | cost = Through SVM, the cost reflects the trade-off between achieving perfect separation of the training data and the degree of some misclassifications. A higher cost value represents the risk of overfitting but few classification errors.  degree = This hyperparameter establishes the decision boundary, with higher values allowing greater flexibility but increasing the risk of overfitting.  scale_factor = This hyperparameter controls the kernel width to ensure data is normalised effectively for optimal performance without altering patterns. | kernlab |
| LASSO | LASSO is a regression technique incorporating regularisation to improve model accuracy and interpretability. By adding a penalty to the loss function, LASSO forces some feature coefficients to become zero, effectively performing variable selection. This variable selection process makes it useful in situations where the dataset has many features, helping to reduce overfitting and enhance the generalisability of the model. | penalty = The penalty hyperparameter in LASSO regression controls the size and magnitude of regression coefficients. This penalty is the sum of the values of the coefficients (L1 norm), encouraging some coefficients to be zero, which then excludes specific predictors from the model. This process reduces overfitting by selecting the most relevant features and simplifies the model. The strength of this penalty, often represented by lambda (λ), is fine-tuned through cross-validation to find the optimal balance between model complexity and predictive performance. | glmnet |
|  | | | |
